# How Beds Are Endowed at Leicester

**Published:** 1921-01-08

**Authors:** 


### MUSIC, THE DRAMA, AND SPORT.

### How Beds are Endowed at Leicester.

IN this year of great financial anxiety it is pleasing to record a succession of successful efforts in Leicester for the Royal Infirmary. A concert organised by Mr. Percy H. Wykes, who has, on many occasions, lent his help to the institution, produced a sum of £400. Mr. Wykes has been elected a Life Governor of the Infirmary in recognition of his great interest and generous support. His election recalled to the Governors an unbroken interest taken by the Wykes family, and more particularly by the late Mr. T. A. Wykes, for forty-one years Secretary of the institution.

A combined effort by the Sunday-school children of the city and county last year, under the Rev. Canon Papprill and the Rev. F. Lansdown, resulted in a contribution of £300 towards the endowment of a "Sunday-school Scholars" cot in the Children's Hospital, and the organisers hope that a renewed effort this Christmas will complete the endowment. The Leicester Amateur Music and Dramatic Society, after recent performances, have sent £100 to the infirmary and £40 to the Children's Hospital, with a donation of £4 4s. from Mr. R. D'Oyly Carte, who wished to recognise the helpful work of the local Society. These efforts have been continued for many years, and by arrangement sums received are accumulated by the institution with a view to the permanent endowment of cots in the Children's Hospital. Two cots have been endowed in this way, and a third is in process of endowment.

A great effort by Mr. Walter Martin and the players associated with him culminated in the complete endowment of a "Walter Martin's Players" cot in the short space of two seasons.

The Palace Matinée Committee again secured the goodwill of Sir Oswald Stoll in the free use of the local theatre, the staff, orchestra, and artists, and their annual effort was again more successful than any previously organised. The Matinée Committee have, up to the present, helped to contribute £2,315 to the funds, and their recent effort will add at least £650.

In Leicester it is in keeping with tradition that the sporting associations should lend substantial assistance to charitable effort. The Leicester Football Club are setting aside a benefit match for the funds of the infirmary; the last match so organised resulted in a contribution of £442. The club has already endowed one bed by means of benefit matches. The Leicester City Football Club have followed the lead of the sister club, and the result of benefit matches has been a sum of £574. The President and officers of the club have the permanent endowment of a bed in view, and intend to give a further benefit match at the close of the season, in the hope of completing the endowment of the "Leicester City Football Club" bed. The Leicester and Leicestershire Bowlers' Association have, for several years, undertaken the annual endowment of one cot in the Children's Hospital, and are raising, in addition, money for the permanent endowment of a cot. A bowlers' annual cot service at the Presbyterian Church gives great impetus to the movement.

A "Leicester Cyclists' Union" cot, which was endowed some years since, adds to the number of cots endowed in association with sport. The combined outcome of music, drama, and sport in Leicestershire is a monument of goodwill to the local hospital.

